# The quantified method for blood clot detection in the extraction socket

**DOI:** 10.12688/f1000research.155330.1

**Published:** 2024-09-10

**Authors:** Suwat Tanya, Piyachat Patcharanuchat, Sajee Sattayut

**Affiliations:** 1 Graduate School, Faculty of Dentistry, Khon Kaen University, Nai Mueang, Khon Kaen, 40002, Thailand; 2 Lasers in Dentistry Research Group, Khon Kaen University, Nai Mueang, Khon Kaen, 40002, Thailand; 3 Department of Preventive Dentistry, Faculty of Dentistry, Khon Kaen University, Nai Mueang, Khon Kaen, 40002, Thailand; 4 Department of Oral and Maxillofacial Surgery, Faculty of Dentistry, Khon Kaen University, Nai Mueang, Khon Kaen, 40002, Thailand

**Keywords:** tooth extraction, blood coagulation, validation study, capillary action, techniques

## Abstract

**Background:**

Currently, there is no objective and quantified measurement for detecting blood clots during extraction socket hemostasis. It has relied solely on clinical observation, even when conducting clinical research by using extraction sockets as samples. This study aimed to assess the in vitro reliability and clinical-relevant validity of a new objective measurement providing quantified data called blood clot detection (BCD) using a standard capillary tube.

**Methods:**

The in vitro part of the study was conducted using surplus blood samples from ten healthy participants. Two identical sets of blood samples in simulation reservoirs mimicking bleeding sockets were prepared for reliability tests. Then, the capillary tubes were concurrently placed in the reservoirs. The blood-filled distances were measured. The part of clinical-relevant validity study was conducted in sixteen extraction sockets from each healthy participant. Clinical observation and BCD measurement were evaluated by two calibrated assessors. The total duration of the assessment was a 30-minute.

**Results:**

The distances of the blood-filled capillary tube were decreased by time. Test and retest reliability analysis of the BCD measurement showed an excellent intraclass correlation coefficient of 0.980 (0.968 to 0.988). The medians of blood-filled distance categorized by clinical observation into active bleeding, sluggish oozing, and clot formation were 13.0 mm (Q1 = 11.7, Q3 = 13.8), 5.6 mm (Q1 = 4.3, Q3 = 7.0), and 0.9 mm (Q1 = 0.5, Q3 = 1.3), respectively. The blood-filled distance of the clot formation group was significantly less than the active bleeding and sluggish oozing (p<0.001). Therefore, the BCD measurement also significantly indicated the completion of extraction socket hemostasis

**Conclusions:**

A distance of blood-filled in capillary tube of 0.9 mm from the BCD measurement significantly ensured complete clot formation. The BCD measurement proved to be a quantified tool for objectively measuring hemostasis of bleeding socket.

## Introduction

Since 1998, the bleeding extraction socket has been widely used as a research model found in PubMed databases for investigating the effectiveness of interventions that enhance hemostasis and for observing patients with bleeding tendencies. ^1^ Forty clinical trials have examined the socket hemostasis following the use of new hemostatic agents such as chitosan dressing, ^2^ platelet-rich fibrin ^3^ and novel gelatin-based sponge ^4^ as well as innovative techniques for hemostasis such as, light-emitting diode. ^5^^,^^6^ These clinical trials conducted in both healthy individuals and patients taking antithrombotic medications. ^1^ To evaluate socket hemostasis, various outcome measurements have been employed in clinical research including investigator observations, ^3^^,^^4^^,^^7^^–^^10^ bleeding time, ^2^^,^^5^^,^^6^^,^^11^ Visual Analog Scale (VAS) ^12^ or patient self-report. ^13^^,^^14^ In 38 out of 40 clinical trials on socket hemostasis evaluation, clinical observation was the most utilized method for confirming clot formation. ^1^ However, these subjective outcome measurements obviously depend on investigator experiences, which may result in unreliable findings. Additionally, in a trial conducted by Yerragudi et al., in 2023, it was observed that healthy patients had an incidence of 8% and 6.8% of post extraction bleeding (PEB) after 10-minute and 60-minute pressure hemostasis, respectively. ^9^ Despite applying pressure for the entire duration of physiologic clotting time and confirming clot formation through investigator observation, PEB was still observed. ^9^^,^^10^ Depending solely on clinical observation method to ensure blood clot formation in the extraction socket may not be sufficient for clinical research. In a recent systematic review conducted by Mahardawi et al., in 2023, it was highlighted that there has been no established standard for outcome measurement of socket hemostasis. ^15^ There were few objective measurements for socket evaluation including qualifying the volume of blood loss following tooth extraction ^8^^,^^16^ and counting the number of gauzes used. ^17^ Nevertheless, these approaches are often impractical due to the time required for result interpretation and the lack of an established clinical reference cut point. Therefore, the development of new objective measurements confirming clot formation that can be easily applied in both dental practice and clinical research is essential.

In order to detect blood clots, it is important to consider the properties of microfluidic diagnostic devices. These devices should be personalized, reliable, and valid biomedical tools that offer benefits such as being affordable, requiring fewer samples to generate results and providing rapid analysis. ^18^ According to these concepts, using a standard capillary tube to assess clinical blood clot of extraction socket, namely blood clot detection (BCD), was initiated. The BCD measurement was developed simply based on capillary action, which was the increase of liquid level in a narrow tube due to molecular attraction between liquid and solid. ^19^^–^^21^ In 1963, capillary tubes were reported as reliable for clotting time testing in medicine but have not been applied in dentistry. ^22^ The classic Lucas-Washburn (LW) equation explained the spontaneous capillary flow of Newtonian liquids. The equation was h ^2^ = (rγt cos θ) / (2μ), where h was the liquid level in the capillary tube, r was the radius of the tube, γ was the surface tension, t was the length of time to fill the capillary tube, θ was the contact angle between the liquid and the surface of the capillary tube, and μ was the viscosity of the liquid. ^23^ Therefore, the blood level in the capillary tube decreased when blood viscosity gradually increased by phase transformation from liquid into gel. ^24^^,^^25^ The recent publications suggested that human blood, though composed of non-Newtonian components, behaved like Newtonian liquids and can be predicted by the LW equation. ^20^^,^^26^

The development of the BCD measurement can greatly benefit dental practice and clinical research. To conduct a clinical study on the BCD measurement in dental practice, it is essential to have a reliable and valid biomedical tool that can objectively detect blood clots in the extraction socket. This study aimed to evaluate the in vitro reliability and clinical-relevant validity of the BCD measurement by using a standard capillary tube in extraction sockets.

## Methods

The BCD measurement tool used a standard capillary tube. When it was immersed into the bleeding socket, the distance of blood-filled capillary tube indicated the completion of blood clotting in the socket. The study was designed to prove the reliability and validity of this clinically objective measurement compared to the clinical observation method, which is commonly used in clinical practice and trials.

### Study design

The non-intervention study consisted of two parts: part I entailed conducting an in vitro reliability test of the BCD measurement using a simulation of a blood reservoir, while part II involved clinically validating the BCD measurement by comparing it with clinical observation.

### Study size

The sample size estimation was based on suggested clinical trial sample sizes for continuous measurement interventions in the absence of previous similar studies. ^27^ In this study, the minimum sample was 10.

### Part I in vitro reliability test of the BCD measurement

**Setting and participant recruitment**

This in vitro study was to assess the reliability of two identical sets of standard capillary tubes placed in microtubes as blood reservoirs. Ten healthy volunteers, who had previously donated blood to the central blood bank at Srinagarind Hospital, Faculty of Medicine, Khon Kaen University, Thailand were purposefully recruited to participate in the study. The data were collected between August and October 2022, following the ethical approval. The study protocols have been reviewed and approved by the Khon Kaen University Ethics Committee for Human Research based on the Declaration of Helsinki and the ICH good clinical practices guideline (No. HE651300 on 3 July 2022). Prior to participation, the blood donors were provided with written informed consent by the assessor (ST).

The inclusion criteria were healthy and literate participants aged 18 to 45 years who had an American Society of Anesthesiologists (ASA) class I status. ^28^ The exclusion criteria were participants with systemic diseases, recent medication use within a month and bleeding tendencies induced by systemic conditions.

**The blood sample preparation and allocation**

The simulation of the blood reservoir was conducted using microtube with a small aperture in the cover to accommodate a 75μl-standard capillary tube (Vitrex ^®^ BRIS micro haematocrit tubes, Vitrex Medical A/S Denmark). Each microtube received 0.1 ml of human whole blood to replicate the conditions within an extraction socket. A total of 14 imitated blood reservoir samples were prepared within 4 minutes to prevent the formation of blood clots.

**Figure 1.**
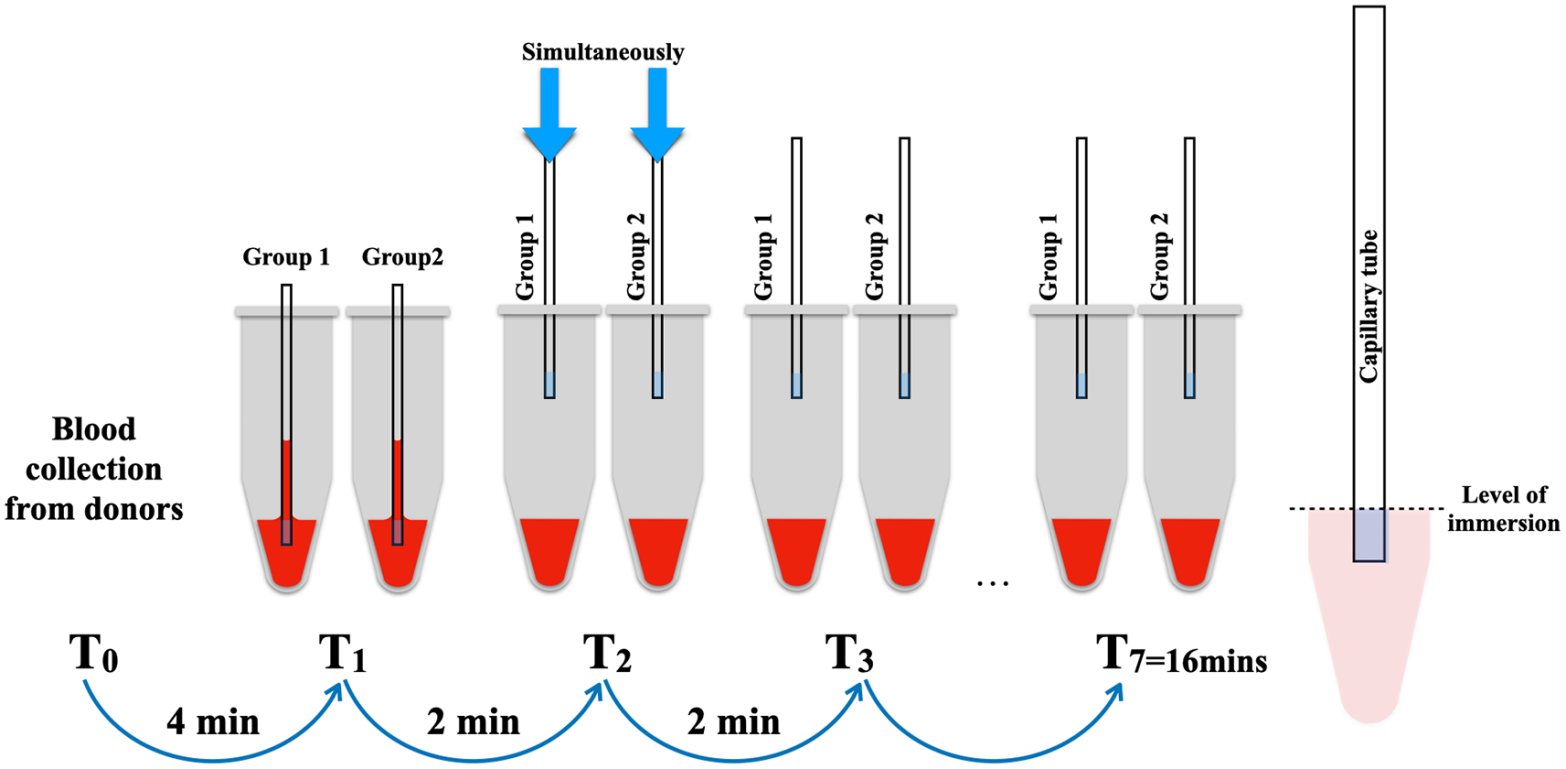
The method of BCD measurement in simulation blood reservoirs mimicking blood oozing in extraction socket.

Subsequently, the 14 microtubes were randomly divided into two groups, with 7 samples allocated to each group: test (group 1) and retest (group 2). The capillary tubes were concurrently immersed into the blood at the level of the blue marker of the capillary tube, with intervals of 2 minutes until the seventh sample of each group. Following immersion, the capillary tubes remained in the blood reservoirs for 10 seconds until the blood level stabilized ( Figure 1). The photographs were captured using a DSLR Canon 90D equipped with a Canon EF 100mm f/2.8L macro lens (Canon, USA) to facilitate the measurement of the length of the blood level in the capillary tube using the ImageJ program (version 1.53) ( Figure 3).

**Distance of blood level in the capillary tube assessment for the BCD measurement**

**Figure 2.**
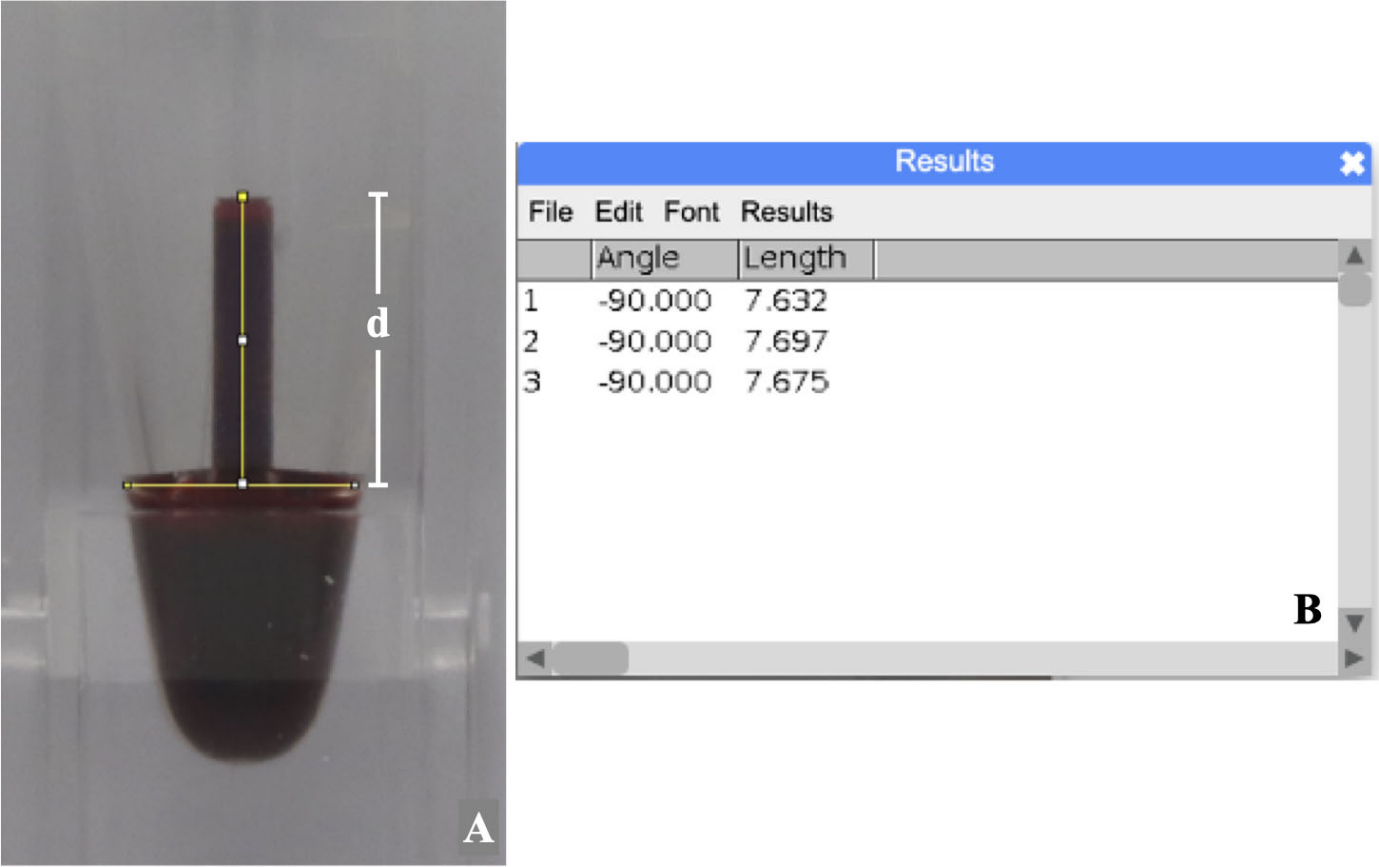
The measurement of blood level from the recorded photograph representing BCD measurement.

The photograph of the blood level in the capillary tube was measured three times using the ImageJ program (version 1.53). The distance (d) in millimeters from the top center of the meniscus to the imaginary line of the lower surface of the blood-filled capillary tube was defined as the blood level ( Figure 2). To calculate the intra-examiner reliability, ten percent of sample photos were randomly selected and assigned a random sequence by a computer-generated program. ^29^ On Day 1, the assessor measured blood levels and repeated the measurement on Day 7 with the same photos.

### Part II clinical-relevant validity of the BCD measurement

In order to prove the clinical validation of the BCD measurement, the comparison between clinical observation and the BCD measurement for assessing blood clot formation after tooth extraction was conducted.

**Setting and participant recruitment**

Inclusion criteria were healthy participants categorized into ASA class I status, ^28^ 18 to 60 years old, literate, and required tooth extraction at The Oral and Maxillofacial Clinic, Faculty of Dentistry, Khon Kaen University, Thailand. Exclusion criteria applied to participants taking antiplatelets, anticoagulants, immunosuppressive drugs, allergic to local anesthesia, with a tendency to bleed from systemic conditions, unwilling to participate, and those with previously treated teeth, acute infections, retained roots, or periapical radiolucency greater than 5-mm in diameter. The criteria for withdrawal were active bleeding after extraction caused by damaged gingival tissue, root fracture during extraction, and the need for surgical removal. The data collection was conducted between March and August 2023, following the ethical approval. The study protocols have been reviewed and approved by the Khon Kaen University Ethics Committee for Human Research based on the Declaration of Helsinki and the ICH good clinical practices guideline (No. HE662004 on 8 February 2023). A total of 16 sockets were included in this particular part of the research.

**Extraction procedure used in this study**

The procedure of tooth removal was a routine extraction. ^30^ Local anesthesia using 2% mepivacaine hydrochloride 20 mg/mL with adrenaline 1:100,000 (Scandonest 2% special, Septodent, France, B32091AC, imported and distributed by Accord Corporation Limited, Thailand) was performed. For mandibular teeth anesthesia, 1.5 ml of local anesthesia was used for the inferior alveolar nerve block including the lingual nerve block. The 0.3 ml of local anesthesia was used for buccal local infiltration as necessary. The maxillary teeth anesthesia was achieved through 0.6 ml of local anesthesia for each supraperiosteal injection and 0.2 ml of local anesthesia for palatal infiltration. Therefore the total amount of local anesthesia used was between 1 and 2 cartridges per case. The extraction was conducted using a standardized routine extraction ^30^ with minimized trauma maneuvers by three calibrated oral and maxillofacial surgeons. After extraction, the socket was gently wiped with sterile gauze and curetted if needed. To validate the BCD measurement using clinical observation, two calibrated assessors (ST and HD) were assigned to assess the extraction socket using different methods. If a participant required multiple extractions, only one extraction socket was selected based on inclusion criteria and prioritized by the sequence of extraction.

### Methods of validating BCD measurement with clinical observation

The BCD measurement was clinically validated alongside clinical observations. The clinical observation criteria were modified from the studies conducted by Nagraj et al. ^11^ and Moran et al. ^31^ The categorized types of socket bleeding were normal bleeding, primary PEB, reactionary PEB, secondary PEB ^11^ and blood oozing. ^31^ In this study, the evaluation was categorized into three groups: “active bleeding” (continuous blood flow without being hemodynamically stable), “sluggish oozing” (blood movement observed but not overflowing), and “clot formation” (no blood flow due to transformation from liquid to solid gel).

The first assessor (HD) conducted a clinical assessment of extraction sockets performed by calibrated oral and maxillofacial surgeons. The observation period lasted 10 seconds. Following this, the second assessor (ST) evaluated the sockets using the BCD measurement by placing a standard capillary tube in contact with the blood surface in the extraction, as was done in part I of this study, also for 10 seconds. Following this, gauze pressure hemostasis was applied for 5 minutes. Both assessments of blood clots were repeated every 5 minutes after the interval pressure hemostasis, with the evaluation time limited to a maximum of 30 minutes. If active bleeding persists beyond this time, the surgeon may consider the utilization of local hemostatic agents.

The data from the clinical observation method was recorded in a data collection form, while the photographs of the blood-filled capillary tube were taken using a DSLR Canon 90D camera (Canon, USA). The distance of blood level in the capillary tube in mm was measured three times using the ImageJ program (version 1.53), ^32^ these measurements were then averaged to determine the average length of blood levels in the capillary tube.

### Variables and measurements

The main outcome of the study was measuring blood length in the capillary tube, referred to as the BCD measurement. This measurement was quantified in millimeters using the ImageJ software to analyze photographic data. Other related outcomes encompassed the duration of blood clot formation and the criteria for clinical assessment.

### Bias

In order to avoid selection bias, the participants were recruited by the health care workers who did not involve in the research team. In part I, the participants were invited by a nurse who was independent of the research team. In part II, the participants, the routine patients undertaken tooth extraction, were invited by oral and maxillofacial surgeons who followed specific inclusion and exclusion criteria.

### Statistical analyses

The data were explored using Shapiro-Wilk tests to assess the normality. For normally distributed data, the intraclass correlation coefficient (ICC) was calculated to determine the intra-examiner reliability. The test and retest reliability were evaluated by comparing the length of blood levels in capillary tubes between the duplicated set of samples; between test and retest groups. For descriptive statistics, continuous data were expressed as the average with their 95% confidence interval (95% CI) and standard deviation (SD), while categorical data were presented as frequency. Repeated measures ANOVA was used to compare blood level lengths in capillary tubes within subjects over a duration of 4 to 16 minutes, with a 2-minute interval.

To compare the distance of blood levels in capillary tubes with clinical observations, ANOVA with Tukey tests was conducted. In cases of non-normal distribution, descriptive statistics were presented using the median (Me), 1 ^st^ quartile (Q1) and 3 ^rd^ quartile (Q3) were presented for descriptive statistics. A non-parametric Kruskal Wallis test with Bonferroni correction were conducted.

All the analyses in this study were performed at a significance level of 0.05 using SPSS program (version 26.0. Armonk, NY: IBM Corp.).

## Results

The results were presented in two parts according to the study methods.

### Results of part I in vitro reliability test of the BCD measurement

In this part of study, there were 5 males and 5 females involved. The demographic data was shown in Table 1. The hemoglobin levels of all the volunteers were within normal limits.

**Table 1.** Demographic data of the participants in part I of this study.

Demographic data	Average (95% CI)	SD	Range
Gender (male = 5, female = 5)			
Age (years)	28.70 (22.94 to 34.46)	8.06	18 to 43
Male	32.60 (22.49 to 42.71)	8.14	22 to 43
Female	24.80 (16.78 to 32.81)	6.56	18 to 35
Hemoglobin (mg/dL)	14.37 (13.66 to 15.08)	1.00	12.5 to 16.2
Male	14.74 (13.64 to 15.84)	0.88	14.0 to 16.2
Female	14.00 (12.68 to 15.32)	1.06	12.5 to 15.2

95% CI – 95% confidence interval; SD - standard deviation.

### Intra-examiner reliability

The Shapiro-Wilk tests revealed that the average lengths of blood in the capillary tube from the recorded photographs measured on Day 1 and Day 7 exhibited normal distributions at p = 0.799 and p = 0.585, respectively. The result of intra-examiner reliability was excellent. The ICC with 95% confidence interval was 0.989 (0.973 to 0.996), at p < 0.001.

### Test and retest reliability of the BCD measurement

Exploring the distribution of the average lengths of blood in capillary tubes of test group and retest group from 10 subjects, the Shapiro-Wilk tests confirmed the normal distribution at p = 0.294 and p = 0.130, respectively. Thus, descriptive statistics including the average with their 95% CI and SD were used to describe the data in Table 2. Over the observation period of 4 to 16 minutes, the average lengths of blood in the capillary tubes of both groups gradually decreased. The test and retest reliability analyses showed an excellent ICC of 0.980, with a 95% CI of ICC from 0.968 to 0.988 and a significance level of p<0.001. Additionally, a repeated measures ANOVA analysis showed no significant difference in the average lengths of blood in the capillary tubes within subject during the observation period (p = 0.770).

**Table 2.** The average blood levels in the capillary tube by the groups.

Time (mins)	Average blood levels (mm) with (95% CI)			
	Group 1 (n=10)	SD	Group 2 (n=10)	SD
4	8.43 (8.06 to 8.80)	0.52	8.31 (7.86 to 8.77)	0.63
6	7.84 (7.46 to 8.21)	0.52	7.77 (7.43 to 8.01)	0.48
8	7.63 (7.20 to 8.07)	0.61	7.47 (7.08 to 7.85)	0.54
10	7.31 (6.80 to 7.80)	0.69	7.18 (6.72 to 7.64)	0.64
12	7.03 (6.47 to 7.61)	0.79	6.96 (6.38 to 7.54)	0.81
14	6.64 (6.20 to 7.08)	0.61	6.69 (6.24 to 7.13)	0.62
16	6.26 (5.78 to 6.74)	0.67	6.25 (5.73 to 6.77)	0.72
ICC (95% CI)	0.980 (0.968 to 0.988)			
df/statistic value	df1=69; df2=69/F = 50.523			
p-value	<0.001			

95% CI – 95% confidence interval; n – number of subjects; SD - standard deviation; ICC - Intraclass Correlation Coefficient; df – degree of freedom.

Based on the findings of this in vitro study, the measurement of BCD using the length of blood in the capillary tube demonstrated consistent repeatability at each specific time point and within the same subject over an extended period.

### Results of part II clinical-relevant validity of the BCD measurement

In part II of the study, 16 extraction sockets were included, involving 7 males and 9 females, as illustrated in Table 3. Each subject underwent a single tooth extraction. There was no evidence of socket or subject withdrawal. The average age was 27.7 years old. Based on the initial examination of vital signs and body mass index, they were within normal limits.

**Table 3.** Demographic data of the participants in part II of this study.

Demographic data	Average (95% CI)	SD	Range
Gender (male = 7, female = 9)			
Socket location (Maxillary socket = 8, Mandibular socket = 8)			
Age (years)	27.7 (21.2 to 34.2)	12.2	19 to 59
BMI (kg/m ^2^)	21.9 (20.2 to 23.5)	3.1	16.7 to 27.3
SBP (mmHg)	120.6 (112.6 to 128.7)	15.1	96 to 139
DBP (mmHg)	73.6 (67.8 to 79.5)	11.1	51 to 94
HR (bpm)	83 (76.2 to 89.9)	12.9	61 to 102

95% CI – 95% confidence interval; BMI - body mass index; SBP - systolic blood pressure; DBP - diastolic blood pressure; HR - heart rate.

### Blood levels in the capillary tube by times

**Figure 3.**
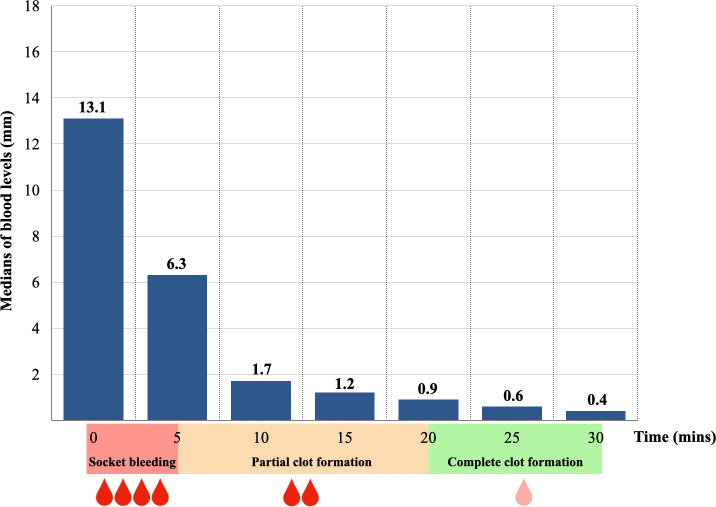
The medians of blood level in the capillary tube by times.

The lengths of blood levels in capillary tubes by times demonstrated a normal distribution according to the Shapiro-Wilk tests, while at 10 minute and at 30 minutes of observation periods, the distributions were not a normal distribution at p = 0.006 and p = 0.023, respectively. Thereby, the medians were used to describe the data. Upon immediate post-extraction assessment, it was observed that only one socket exhibited sluggish oozing, while the remaining sockets were classified as active bleeding sites. The correspondence between the medians of blood levels based on the BCD measurement and the clinical observation over the time of assessment was illustrated in Figure 3. Following a 5-minute of pressure hemostasis, a marked reduction in the medians of blood levels from 13.1 mm (Q1 = 11.8, Q3 = 14.7) to 6.3 mm (Q1 = 2.3, Q3 = 10.7) were observed. It decreased gradually from 1.7 mm (Q1 = 0.9, Q3 = 3.0) at 10 minutes to 1.2 mm (Q1 = 0.5, Q3 = 1.9) at 15 minutes. After a 20-minute of pressure hemostasis, all the sockets had complete clot formation and the medians of blood levels decreased slightly from 0.9 mm (Q1 = 0.4, Q3 = 1.2) to 0.6 mm (Q1 = 0.3, Q3 = 0.8) at 25 minutes and 0.4 mm (Q1 = 0.0, Q3 = 0.6) at the end of the observation.

### Blood levels in the capillary tube by clinical observation

The Shapiro-Wilk tests demonstrated the evidence of normality of the length of blood level in active bleeding and sluggish oozing categories at p = 0.715 and p = 0.155, respectively, while in clot formation category exhibited a non-normal distribution (p <0.001). Therefore, descriptive statistics including the median, Q1 and Q3 were used to describe the data in Table 4. A Kruskal-Wallis test with Bonferroni correction showed a significant difference in the median of blood levels among clot formation sockets compared to both active bleeding and sluggish oozing sockets ( Table 4, 5). Based on the finding of the BCD measurement, 0.9 mm of blood-filled capillary tube corresponded with complete clot formation in extraction socket.

**Table 4.** The median blood levels in the capillary tube categorized by the clinical observation.

	Clinical observation (frequency=112)	Me	Q1	Q3	Range	df	Kruskal-Wallis test (statistic value)	p-value
BCD measurement	Active bleeding (21)	13.0 ^a^	11.7	13.8	7.8 to 21.4	2	67.229	<0.001
	Sluggish oozing (11)	5.6 ^a^	4.3	7.0	1.1 to 8.9			
	Clot formation (80)	0.9 ^b^	0.5	1.3	0.0 to 3.08			

Me-Median; Q1-1 ^st^ quartile; Q3-3 ^rd^ quartile; df – degree of freedom; ^a,b^ The same letters showed no significant difference.

**Table 5.** The comparisons of the blood levels among the groups categorized by blood formation in clinical observation.

	Statistic value	Standard error	p-value
Clot formation -sluggish oozing	-43.15	10.439	0.000
Clot formation -active bleeding	-60.864	7.959	0.000
Sluggish oozing-active bleeding	-17.714	12.082	0.428

## Discussion

Based on our findings from part I of the study, we confirmed that the BCD measurement was reliable in vitro. There was an excellent Intraclass Correlation Coefficient (ICC) between two identical groups. The blood level in the capillary tube decreased due to an increase in blood viscosity caused by physiologic clot formation. ^24^^,^^25^ This evidence aligns with the LW equation. ^23^ In this study, the blood level was measured from a recorded photograph using ImageJ program, which is a user-friendly freeware for scientific image analysis. ^32^ This approach has helped to reduce measurement error and is suitable for clinical research.

In part II of this study, it was clinically substantiated that the coagulation duration of 18 extraction sockets aligned within the normal range for physiological coagulation time ^33^ in healthy individuals. Following 5 to 15 minutes of clinical scrutiny, a complete blood clot was evident in the majority of extraction sockets and no active bleeding was detected in any socket after 10 minutes of pressure hemostasis. These observations are consistent with the findings of Kumar et al. ^10^ and Yerragudi et al., ^9^ which propose that 10 minutes of pressure hemostasis is sufficient to initiate blood clotting in extraction sockets. Complete clot formation in all extraction sockets was observed following 20 minutes of pressure hemostasis, a duration falling within the recommended range of 30 to 60 minutes in clinical practice post-extraction. ^30^^,^^34^^,^^35^

It is noteworthy that as per Yerragudi et al., ^9^ healthy patients may experience a 6.8% incidence of post-extraction bleeding (PEB) subsequent to 60 minutes of pressure hemostasis, potentially due to inadequate evaluation of socket hemostasis. To mitigate PEB, it is imperative to ensure complete clot formation in the extraction socket before discharging the patient. The length of the blood level in the capillary tube, known as BCD measurement, corresponds to the clinical observation of blood clot formation. Therefore, the BCD measurement provides a reliable tool for objectively indicating socket hemostasis. The average blood level indicating complete clot formation in the extraction socket was approximately 1 mm in the capillary tube, as confirmed by observing clot formation during 20 minutes of pressure hemostasis.

The BCD measurement is a reliable tool that can be utilized in both research and clinical settings. Additionally, it can objectively confirm the formation of a clot in the extraction socket before the patient is discharged. By ensuring proper socket hemostasis, the BCD measurement may assist operators in reducing bleeding problems in patients taking medications. The quantitative data obtained from BCD measurement provide comparable information in clinical research. Its application is also simple for use by general dental professionals in assessing socket hemostasis more accurately than clinical observation alone.

The BCD measurement can confirm the completion of blood clots and the trend of hemostasis in the extraction socket by the length of blood level in the capillary tube. However, it cannot differentiate between active bleeding and sluggish oozing.

In clinical practice, dentists can estimate approximately 1 mm of blood level from the capillary tube through observation to confirm complete blood clot formation. The clinical application of the BCD measurement necessitates cautious consideration due to its reliance on data obtained solely from healthy participants.

## Conclusion

The BCD measurement using a standard capillary tube was found to be reliable and valid for objectively confirming hemostasis in the extraction socket. The distance of the blood level in this measurement indicated trends of blood clot formation in the bleeding extraction socket. Approximately 1 mm of blood level from the BCD measurement significantly corresponds to complete blood clot formation in clinical observation.

### Ethical considerations

The Khon Kaen University Ethics Committee for Human Research approved the study protocols based on the Declaration of Helsinki and the ICH good clinical practices guideline (No. HE651300 for in vitro reliability test and No. HE662004 for clinical-relevant validity study). All participants provided written informed consent.

## Ethics and consent

The Khon Kaen University Ethics Committee for Human Research approved the study protocols based on the Declaration of Helsinki and the ICH good clinical practices guideline (No. HE651300 for in vitro reliability test and No. HE662004 for clinical-relevant validity study). All participants provided written informed consent.

## Data Availability

Figshare: The quantified method for blood clot detection in the extraction socket. https://doi.org/10.6084/m9.figshare.26780050.v2. ^36^ This project contains the following underlying data:
- Distance of blood-filled capillary tube in part I of the study.xlsx - Distance of blood-filled capillary tube and clinical observation in part II of the study.xlsx The corresponding author provided the underlying data upon request to ensure transparency in conducting human research, as requested by the journal. This complies with the regulations of the Khon Kaen University Ethics Committee for Human Research, and has been done with the consent of the subjects involved in this study. Figshare: STROBE checklist of of the quantified method for blood clot detection in the extraction socket. https://doi.org/10.6084/m9.figshare.26531323.v1. ^37^ This project contains the following:
• STROBE checklist Data are available under the terms of the Creative Commons Attribution 4.0 International license (CC-BY 4.0).
