# Supplementary material for: The quantified method for blood clot detection in the extraction socket
Source: F1000Res. 2025 Apr 7;13:1043. Originally published 2024 Sep 10. [Version 2] doi: 10.12688/f1000research.155330.2 (PMC11914871; doi:10.12688/f1000research.155330.2)
Supplement: Peer review history

# Reviewer report: Reviewer response for version 2

**DOI:** 10.5256/f1000research.180062.r376382

**Published:** 2025-04-16

**Authors:** Christian Khoswanto

**Affiliations:** 1 Department of Oral Biology Faculty of Dentistry, Airlangga University, Surabaya, Indonesia

I have no further comments to make.

Is the work clearly and accurately presented and does it cite the current literature?

Partly

If applicable, is the statistical analysis and its interpretation appropriate?

Yes

Are all the source data underlying the results available to ensure full reproducibility?

Yes

Is the study design appropriate and is the work technically sound?

Partly

Are the conclusions drawn adequately supported by the results?

Partly

Are sufficient details of methods and analysis provided to allow replication by others?

Partly

Reviewer Expertise:

wound healing

I confirm that I have read this submission and believe that I have an appropriate level of expertise to confirm that it is of an acceptable scientific standard.

# Reviewer report: Reviewer response for version 1

**DOI:** 10.5256/f1000research.170492.r367635

**Published:** 2025-03-17

**Authors:** Yani Corvianindya Rahayu

**Affiliations:** 1 Department of Oral Biology, Faculty of Dentistry, University of Jember, Jember, East Java, Indonesia

Overall this article was written exceptionally well, and** the research method can follow clearly, so that easy for other researchers to reproduce the work.

 This research is quite innovative because there are not many measuring instruments used to measure blood cloth accurately. The development of the BCD method is expected to provide benefits in the field of clinical dentistry.

 There are several things that may need attention:

 1. The detection method is closely related to the measuring instrument, it would be good to be conveyed in detail in the material and method.

 2. In the subjects measured there was a fairly wide age range (18-60 years), which could possibly affect the results. Because in old age there is generally a decrease in hormone function and response to local inflammation, which can have an impact on the formation of blood clots. However, this can be studied in further research.

Is the work clearly and accurately presented and does it cite the current literature?

Partly

If applicable, is the statistical analysis and its interpretation appropriate?

Yes

Are all the source data underlying the results available to ensure full reproducibility?

Yes

Is the study design appropriate and is the work technically sound?

Yes

Are the conclusions drawn adequately supported by the results?

Partly

Are sufficient details of methods and analysis provided to allow replication by others?

Partly

Reviewer Expertise:

Oral Biology, Oral wound healing, Tissue regeneration.

I confirm that I have read this submission and believe that I have an appropriate level of expertise to confirm that it is of an acceptable scientific standard.

## References

[1] : The quantified method for blood clot detection in the extraction socket. *F1000Research* .2024;13: 10.12688/f1000research.155330.1 10.12688/f1000research.155330.1 PMC1191487140104090

# Reviewer report: Reviewer response for version 1

**DOI:** 10.5256/f1000research.170492.r367634

**Published:** 2025-03-11

**Authors:** Olivia Avriyanti Hanafiah

**Affiliations:** 1 Department of Oral and Maxillofacial Surgery, Faculty of Dentistry, University of Sumatera Utara, North Sumatra, Indonesia

This study demonstrates that BCD exhibits high repeatability; however, it does not establish that BCD is more accurate or superior to clinical observation. In the final section of the discussion, the authors state, “Its application is also simple for use by general dental professionals in assessing socket hemostasis more accurately than clinical observation alone.” The claim of “more accurately” is not substantiated by the study design or statistical analysis, as clinical observation was still used as the gold standard for validation. If the objective was to demonstrate that BCD is more accurate than clinical observation, a comparison with a more objective method for assessing complete clot formation (e.g., molecular examination) would be necessary.

 Although the in vitro study indicates a measurable trend in blood coagulation using capillary tubes, there is no evidence that this method provides a clinical advantage over conventional observation. The introduction highlights the limitations of clinical observation—where clot formation may appear complete, yet post-extraction bleeding (PEB) may still occur. However, this study does not establish whether BCD can better ensure complete clot formation. Given the study design, this question remains unanswered, as clinical observation was used as the validation reference rather than serving as a comparator in a superiority analysis. Furthermore, no observations of PEB were included in the study. A more appropriate conclusion would be that BCD produces results “comparable to clinical observation” rather than being “more accurate.” The authors should also exercise caution in their interpretation, as the introduction characterizes clinical observation—used as the validation reference—as a subjective method with potentially unreliable outcomes.

 To enhance replicability and methodological transparency, the following details should be included in the methods section:

 1. The in vitro methods section states that the blood reservoir was prepared within four minutes to prevent clot formation. A reference should be provided to support the claim that clotting does not occur within four minutes under the experimental conditions applied in this study.

 2. The immersion limit is described only as “up to the blue line” without specifying an exact measurement in millimeters. This could be problematic if the position of the blue line varies across different capillaries or if future studies use capillaries from different manufacturers.

 3. The capillary tube specifications (diameter and length) are not provided. These details are crucial, as excessive tube length may render the method impractical for clinical application in the oral cavity. Additionally, the diameter influences blood capillarity, making it essential to include complete information to ensure replicability.

 4. No visual documentation is provided regarding socket conditions or the BCD examination procedure. Clinical photographs would be highly beneficial in clarifying the application of this method (e.g., whether the tube is positioned vertically or at an angle, particularly for posterior teeth) and ensuring consistency in evaluation.

 5. The manuscript could improve by providing more details on potential confounders that may have influenced blood clot formation, such as intraoperative trauma or patient-specific factors like platelet function. Moreover, the study lacks a control group, which would strengthen the validation process. The authors should explain how they accounted for potential variability in clotting dynamics between different individuals and different extraction sites.

Is the work clearly and accurately presented and does it cite the current literature?

Partly

If applicable, is the statistical analysis and its interpretation appropriate?

Partly

Are all the source data underlying the results available to ensure full reproducibility?

Partly

Is the study design appropriate and is the work technically sound?

Partly

Are the conclusions drawn adequately supported by the results?

Partly

Are sufficient details of methods and analysis provided to allow replication by others?

Partly

Reviewer Expertise:

Wound healing, Oral wound healing, Biology molecular, Oral and Maxillofacial Surgery

I confirm that I have read this submission and believe that I have an appropriate level of expertise to confirm that it is of an acceptable scientific standard, however I have significant reservations, as outlined above.

# Response

**Published:** 2025-03-11

**Authors:** Sajee Sattayut

**Affiliations:** Khon Kaen University, Thailand

Thank you very much for your suggestion. We will write more details about the capillary and will consider the issue of comparing the BCD and conventional observation techniques.

 Regarding postoperative bleeding, there was no postoperative bleeding in this study.

# Response

**Published:** 2025-03-29

**Authors:** Sajee Sattayut

**Affiliations:** Khon Kaen University, Thailand

Thank you for your comments; all of them were considered to modify our article with more details as follows:

**Comment**

 This study demonstrates that BCD exhibits high repeatability; however, it does not establish that BCD is more accurate or superior to clinical observation. In the final section of the discussion, the authors state, “Its application is also simple for use by general dental professionals in assessing socket hemostasis more accurately than clinical observation alone.” The claim of “more accurately” is not substantiated by the study design or statistical analysis, as clinical observation was still used as the gold standard for validation. If the objective was to demonstrate that BCD is more accurate than clinical observation, a comparison with a more objective method for assessing complete clot formation (e.g., molecular examination) would be necessary.

 Although the in vitro study indicates a measurable trend in blood coagulation using capillary tubes, there is no evidence that this method provides a clinical advantage over conventional observation. The introduction highlights the limitations of clinical observation—where clot formation may appear complete, yet post-extraction bleeding (PEB) may still occur. However, this study does not establish whether BCD can better ensure complete clot formation. Given the study design, this question remains unanswered, as clinical observation was used as the validation reference rather than serving as a comparator in a superiority analysis. Furthermore, no observations of PEB were included in the study. A more appropriate conclusion would be that BCD produces results “comparable to clinical observation” rather than being “more accurate.” The authors should also exercise caution in their interpretation, as the introduction characterizes clinical observation—used as the validation reference—as a subjective method with potentially unreliable outcomes.

**Reply;**

 Thank you for your thoughtful recommendations. In order to follow your suggestion on not over claim the novel technique of measurement. There are some modifications as follows:-

**Revision;**

 “Its application is also simple for use by general dental professionals in assessing socket hemostasis, enhancing more accuracy when combined with standard clinical observation”

**Comment**

 To enhance replicability and methodological transparency, the following details should be included in the methods section:

 1. The in vitro methods section states that the blood reservoir was prepared within four minutes to prevent clot formation. A reference should be provided to support the claim that clotting does not occur within four minutes under the experimental conditions applied in this study.

**Reply;**

 Thank you for highlighting this important aspect. The four-minute timeframe was the minimal period required to collect surplus blood from the main reservoir and prepare 14 replicated samples using a disposable syringe with an 18-gauge needle. The absence of clotting within this timeframe under the experimental conditions was verified by inverting the main reservoir; the lack of a retained solid clot indicated that clot formation had not occurred. Further details are provided as follows:

**Revision;**

 “A total of 14 imitated blood reservoir samples were prepared within 4 minutes using a disposable syringe with an 18-gauge needle. The absence of clotting under these experimental conditions was confirmed by inverting the main reservoir, where the lack of a retained solid clot indicated that blood clot had not occurred."

**Comment**

 2. The immersion limit is described only as “up to the blue line” without specifying an exact measurement in millimeters. This could be problematic if the position of the blue line varies across different capillaries or if future studies use capillaries from different manufacturers.

**Reply;**

 Thank you for your insightful feedback.

 The distance from the edge of the capillary tube to the endpoint of the blue marking was 2 mm. he following are the additional details:

**Revision;**

 “The capillary tubes were concurrently immersed into the blood at the level of the blue marker of the capillary tube, with intervals of 2 minutes until the seventh sample of each group. The distance from the capillary tube’s edge to the endpoint of the blue marking was 2 mm.”

**Comment**

 3. The capillary tube specifications (diameter and length) are not provided. These details are crucial, as excessive tube length may render the method impractical for clinical application in the oral cavity. Additionally, the diameter influences blood capillarity, making it essential to include complete information to ensure replicability.

**Reply;**

 Thank you for your insightful feedback. The supplementary details are as follows:

**Revision;**

 “The simulation of the blood reservoir was conducted using microtube with a small aperture in the cover to accommodate a 75μl-standard capillary tube. This standard tube had an inner diameter of 1.15 ± 0.005 mm and a length of 75 ± 0.5 mm (Vitrex® BRIS micro hematocrit tubes, Vitrex Medical A/S, Denmark).”

**Comment**

 4. No visual documentation is provided regarding socket conditions or the BCD examination procedure. Clinical photographs would be highly beneficial in clarifying the application of this method (e.g., whether the tube is positioned vertically or at an angle, particularly for posterior teeth) and ensuring consistency in evaluation.

**Reply;**

 We appreciate your keen observation. This study did not provide clinical photographs under the consent and procedure approved by the Khon Kaen University Ethics Committee for Human Research to minimize additional procedures from the routine extraction in this case.

 The capillary positioning was detailed as follows;

**Revision;**

 “Following this, the second assessor (ST) evaluated the sockets using the BCD measurement by placing a standard capillary tube in contact with the blood surface in the extraction, as was done in part I of this study, also for 10 seconds. The tube was positioned parallel to the long axis of the extracted tooth or at an angle between 45 to 90 degrees, particularly for lower posterior teeth.”

**Comment**

 5. The manuscript could improve by providing more details on potential confounders that may have influenced blood clot formation, such as intraoperative trauma or patient-specific factors like platelet function. Moreover, the study lacks a control group, which would strengthen the validation process. The authors should explain how they accounted for potential variability in clotting dynamics between different individuals and different extraction sites.

**Reply;**

 Thank you for bringing this key detail to our attention.

 As far as the potential confounders are concerned, we are aware of them by including only healthy participants and excluding those with systemic conditions inducing bleeding tendency. The withdrawal criteria influencing on blood clot formation after extraction, such as active bleeding caused by damaged gingival tissue, root fracture during extraction, and need for surgical removal, were mentioned in this article. All participants showed the reflection of homogeneity exhibited clotting into the extraction socket within the normal bleeding time of 10 minutes of pressure hemostasis. 

 Regarding the control group, this design of the observational method of hemostasis was used as a control method compared with the novel technique of blood clot detection.

# Reviewer report: Reviewer response for version 1

**DOI:** 10.5256/f1000research.170492.r367642

**Published:** 2025-02-25

**Authors:** Christian Khoswanto

**Affiliations:** 1 Department of Oral Biology Faculty of Dentistry, Airlangga University, Surabaya, Indonesia

- In this study, the author did not examine the peripheral blood of patients who would have their teeth extracted, which gave the impression that the samples taken were not homogeneous.

 - It is necessary to add information on whether the samples used were free from congenital systemic diseases.

 - The author needs to add criteria for teeth that were extracted, whether due to caries, loose teeth, or tooth extraction due to orthodontic treatment

Is the work clearly and accurately presented and does it cite the current literature?

Partly

If applicable, is the statistical analysis and its interpretation appropriate?

Yes

Are all the source data underlying the results available to ensure full reproducibility?

Yes

Is the study design appropriate and is the work technically sound?

Partly

Are the conclusions drawn adequately supported by the results?

Partly

Are sufficient details of methods and analysis provided to allow replication by others?

Partly

Reviewer Expertise:

wound healing

I confirm that I have read this submission and believe that I have an appropriate level of expertise to confirm that it is of an acceptable scientific standard, however I have significant reservations, as outlined above.
